# Supplementary material for: Comparative multiomics analysis of cell physiological state after culture in a basket bioreactor
Source: Sci Rep. 2022 Nov 23;12:20161. doi: 10.1038/s41598-022-24687-4 (PMC9686226; doi:10.1038/s41598-022-24687-4)
Supplement: Supplementary file 1 — Supplementary Information 1. [file 41598_2022_24687_MOESM1_ESM.zip › raw data/Proteomic raw data/6.KEGGenrichment/EG.vs.CG/DOWN/pathway/src/00600.html]

map00600


- K12348, K12349, 3.5.1.23, R06518"

- Down regulated proteins

A0A0D9RVZ2(Description:Ceramidase;Gene:ASAH1;log2FC:-1.3699)

- K12348, K12349, 3.5.1.23, R06528"

- Down regulated proteins

A0A0D9RVZ2(Description:Ceramidase;Gene:ASAH1;log2FC:-1.3699)

- K01117, K12350, K12351, K12352, K12353, K12354, 3.1.4.12, R02541"

- Down regulated proteins

A0A0D9QWC2(Description:Sphingomyelin phosphodiesterase;Gene:SMPD1;log2FC:-1.6416)


A0A0D9QVQ6(Description:Sphingomyelin phosphodiesterase;Gene:SMPD3;log2FC:-1.5450)

- K01080, 3.1.3.4, R06522"

- Down regulated proteins

A0A0D9S760(Description:Phospholipid phosphatase 3;Gene:PLPP3;log2FC:-1.3288)

- K01080, 3.1.3.4, R06521"

- Down regulated proteins

A0A0D9S760(Description:Phospholipid phosphatase 3;Gene:PLPP3;log2FC:-1.3288)

- K01080, 3.1.3.4, R06520"

- Down regulated proteins

A0A0D9S760(Description:Phospholipid phosphatase 3;Gene:PLPP3;log2FC:-1.3288)

- K01441, K12348, K12349, 3.5.1.23, R01494"

- Down regulated proteins

A0A0D9RVZ2(Description:Ceramidase;Gene:ASAH1;log2FC:-1.3699)

Close
